# Oculofaciocardiodental syndrome caused by a novel *BCOR* variant

**DOI:** 10.1038/s41439-023-00244-x

**Published:** 2023-06-12

**Authors:** Tomoyo Yamashita, Junko Hotta, Yukiko Jogu, Eri Sakai, Chie Ono, Haruka Bamba, Hisato Suzuki, Mamiko Yamada, Toshiki Takenouchi, Kenjiro Kosaki, Tohru Yorifuji, Takashi Hamazaki, Toshiyuki Seto

**Affiliations:** 1 Department of Medical Genetics, Osaka Metropolitan University Graduate School of Medicine, Osaka, Japan; 2 Department of Pediatrics, Osaka Metropolitan University Graduate School of Medicine, Osaka, Japan; 3 Center for Medical Genetics, Keio University School of Medicine, Tokyo, Japan; 4 Department of Pediatrics, Keio University School of Medicine, Tokyo, Japan; 5 Division of Pediatric Endocrinology and Metabolism, Children’s Medical Center, Osaka City General Hospital, Osaka, Japan

**Keywords:** Rare variants, Development

## Abstract

Oculofaciocardiodental syndrome is caused by variants in the BCL6 corepressor (BCOR) gene. We identified a novel heterozygous frameshift variant, NM_001123385.2(BCOR):c.2326del, that arose de novo in a Japanese girl with characteristic facial features, congenital heart disease, bilateral syndactyly of toes 2 and 3, congenital cataracts, dental abnormalities, and mild intellectual disability. Reports of *BCOR* variants are rare, and further case accumulation is warranted.

Oculofaciocardiodental (OFCD) syndrome (MIM#300166), also called microphthalmia syndromic 2, is caused by variants in the BCL6 corepressor (BCOR) gene located at Xp11.4 and has male-lethal, X-linked, dominant inheritance^1^. Females with OFCD syndrome have skewed X-inactivation, and inheritance between mother and daughter has been reported^2^, but most cases are sporadic^3^. Although the number of reports of OFCD syndrome has been increasing in recent years with the development of next-generation sequencing, only 98 cases of *BCOR* variants are reported in The Human Gene Mutation Database 2022.4 (https://www.hgmd.cf.ac.uk/ac/all.php). Therefore, OFCD syndrome is relatively rare.

OFCD phenotypes are extremely diverse and include congenital or early-onset cataracts; microphthalmia; characteristic facial features (long face, broad nasal tip, arched eyebrows); atrial or ventricular septal defects; dental abnormalities (delayed eruption of primary or secondary teeth, fused teeth); skeletal abnormalities, such as hammer toe and syndactyly; and developmental delay^3^. We diagnosed a Japanese girl with OFCD syndrome caused by a novel heterozygous frameshift variant in *BCOR*.

The patient was a 3-year and 8-month-old Japanese girl. She was born at 40 weeks and 6 days of gestation through normal spontaneous delivery and had a birth weight of 3,352 g. Her developmental milestones were mildly delayed, with head control at 5 months; rolling over at 7 months; sitting without support at 1 year; crawling at 1 year, 6 months; and independent walking at 2 years, 6 months. Her language development was also mildly delayed, with significant language at 2 years. Her parents were nonconsanguineous, and there was no family history of congenital anomalies. From birth, skeletal abnormalities of the limbs, congenital heart disease, and congenital cataracts were observed. She underwent surgery at 10 months of age for an atrial septal defect and patent ductus arteriosus. She underwent lens removal and was supplied with contact lenses for congenital cataracts. She was referred to us at 2 years and 4 months of age for comprehensive genetic analysis.

At her first visit, the patient was 87.7 cm (−0.6 SD) tall, weighed 10.5 kg (−1.4 SD) and had a head circumference of 49.0 cm ( + 0.7 SD). There were no abnormalities in the thorax and abdomen. Her facial appearance and the findings from her oral and limb examinations are shown in Fig. 1a–e. Left radioulnar synostosis (Fig. 1f) and left coronal suture fusion (Fig. 1g) were observed on imaging. Head magnetic resonance imaging showed no abnormal findings, with no ventricular enlargement or cortical dysplasia. She had mild intellectual disability, with an overall Development Quotient of 60 on the Kyoto Scale of Psychological Development. Conventional chromosomal G-banding revealed a normal female karyotype (46, XX). Microarray chromosome analysis (comparative genomic hybridization) showed no pathological copy number variants. Testing for Cornelia de Lange syndrome showed no pathological variants in *NIPBL*, *SMC1A*, *RAD21*, *SMC3*, or *HDAC8*.

**Fig. 1:**
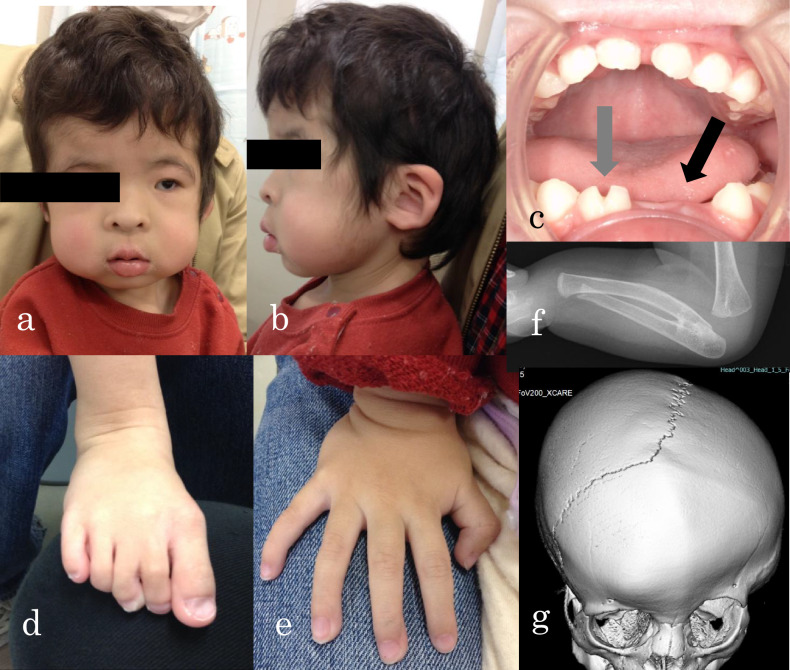
OFCD syndrome clinical phenotypic features. **a**, **b** Facial appearance at 2 years and 4 months old (long face; thick, and downward-curving eyebrows; hypertelorism; exotropia; broad nasal tip; flat nasal root; superior crus of antihelix; small to absent antitragus; low-set ears; full cheeks; high palate; plagiocephaly). **c** Oral findings: abnormal dentition with fused teeth (gray arrow) and congenital absence (black arrow). **d**, **e** Limb findings: bilateral syndactyly of the second and third toes, large hallux, joint flexion contracture, fifth finger clinodactyly. **f** Elbow X-ray showing left radioulnar synostosis. **g** Head CT showing left coronal suture fusion.

For precise diagnosis, this patient was enrolled in the research project “Initiative on Rare and Undiagnosed Diseases (IRUD),” which was conducted in accordance with the Declaration of Helsinki and approved by the ethics committee of our institution. After obtaining informed consent from her parents, we performed whole-exome sequencing (WES). When the patient was 3 years and 8 months old, a heterozygous de novo variant, NM_001123385.2(BCOR):c.2326del;p.(His776Ilefs*10), was identified; this variant has not been reported previously. We confirmed the sequence of BCOR exon 4 by using the direct sequencing method. This variant is absent from control populations (gnomAD. https://gnomad.broadinstitute.org/. Accession 2023/2). The CADD score (https://cadd.gs.washington.edu/snv)^4^ was 28.6. Mutation Taster (www.mutationtaster.org)^5^ predicted the variant as disease causing. Frameshift variants in this downstream region are pathogenic. According to the American College of Medical Genetics and Genomics (ACMG) guidelines, we judged PVS1, PS2, PM2, PM4, PP3 and PP4 to be pathogenic. The IRUD committee at our hospital determined that this novel variant is pathogenic and causes OFCD syndrome.

On the basis of the phenotypic overlap of *BCOR* variants, it has been proposed that OFCD syndrome and one variant of Lenz microphthalmia (MIM #300485) are allelic disorders^1^. Hemizygous men with missense or splice variants in *BCOR* have Lenz microphthalmia syndrome, whereas heterozygous women with a null allele (protein-truncating variants and partial or whole-gene deletion variants) have OFCD syndrome^3^. Ragge et al.^3^ summarized the phenotypes of women presenting with OFCD syndrome in 85 patients from 58 families. The mutations were frameshift variants in 48% of patients and nonsense variants in 33%, with familial inheritance in 26% and sporadic occurrence in 74%. The phenotype was also quite diverse^3^: 82% had finger abnormalities, 13% had radioulnar synostosis, 10% had mild developmental delay, and 9% had hearing loss, in addition to the facial, cranial, nasal, ear, and dental abnormalities and congenital heart disease commonly reported. The phenotype in this case was consistent with that in previous reports (Table 1). Radiculomegaly is also a characteristic feature of OFCD syndrome^6^, but this feature was difficult to evaluate at our patient’s age because it is seen in the secondary teeth. The abovementioned reports suggest that congenital cataracts, congenital atrial septal defects, dental abnormalities, and skeletal abnormalities, including syndactyly of toes 2 and 3, are characteristic findings in OFCD syndrome and that evaluation for a *BCOR* variant is necessary when these signs are present.

**Table 1:** Frequency of inheritance, type of pathogenic variant, and phenotypic characteristics in our patient and in previous reports of patients with OFCD.

	Our patient	Ragge et al.^3^	Redwood et al.^9^
Inheritance	De novo	15/58^a^	4/8
Frameshift/pathogenic variant	Frameshift	28/58^a^	2/10
Congenital cataract	Bilateral	82/83	10/10
Microphthalmia	None	56/83	3/10
Skeletal abnormalities	Bilateral syndactyly of toes 2 and 3, large halluxes and fifth finger clinodactyly	36/67	4/10
Cardiac anomalies	ASD, PDA and PH	51/61	5/10
Dental abnomalies	Fused teeth and congenital absence	68/72	6/10
Development (normal growth and normal deveopmental milestones)	DQ 60	10/16	9/10

Other numbers indicate numbers of cases for which information could be collected.

*ASD* atrial septal defect, *PDA* patent ductus arteriosus, *PH* pulmonary hypertension, *DQ* developmental quantient (Kyoto Scale of Psychological Development).

^a^Number of families for which information could be collected.

Danda et al.^7^ reported two affected sisters with the same nonsense variant in *BCOR*, but the parents were not symptomatic and did not have the variant. This report had an important effect on the genetic counseling of OFCD families, suggesting that the risk of occurrence in a subsequent child is not negligible, even if the mother does not have the variant. On the basis of previous reports of mosaic cases^3,8^, Redwood et al.^9^ suggested that the risk of the mother being somatic or gonadal mosaic for this condition may be higher than for other genetic diseases. For this reason, genetic counseling for the family regarding recurrence was carefully considered and implemented in this case.

The BCL-6 corepressor is a POZ/zinc finger transcriptional repressor that is required for germinal center formation and may also influence apoptosis^10^. The BCoR gene encodes at least two proteins, a long form containing 1721 amino acids (BCoR) and a short form containing 1004 amino acids (BCoR-S). The full-length BCOR protein, but not the shorter version, functions as a corepressor when attached to promoter DNA and enhances BCL6 repression when overexpressed; BCoR-S can interact with BCL-6 and histone deacetylases (HDACs) but weakly represses transcription when in proximity to DNA and does not potentiate BCL-6 repression. Both types of BCOR variants interact with classes I and II of HDAC^10^. Thus, BCOR may functionally link these two classes of HDACs, and histone/protein deacetylation may be the mechanism for BCOR-mediated repression. BCOR is thought to play an important role in the early embryonic development of many body systems^11^. BCL6 is also considered an important oncogene for B-cell development and carcinogenesis, and *BCOR* variants have been identified in tumors such as extranodal NK/T-cell lymphoma and secondary acute myeloid leukemia^12^. Furthermore, *BCOR* in mouse T lymphocytes is likely to be a tumor suppressor^13^. However, the mechanism by which *BCOR* variants cause eye and other abnormalities in OFCD syndrome remains to be elucidated.

In summary, we identified a novel pathogenic variant, NM_001123385.2(BCOR):c.2326del, in a genetically undiagnosed 3-year and 8-month-old girl with congenital cataracts, atrial septal defect, dental anomalies, and syndactyly of toes 2 and 3, and we diagnosed the girl with OFCD syndrome. These eye, heart, dental, and skeletal abnormalities are considered characteristic of OFCD syndrome. Even in de novo cases, careful genetic counseling is required because of the possibility of recurrence in the next child.

## Data Availability

The relevant data from this Data Report are hosted at the Human Genome Variation Database at 10.6084/m9.figshare.hgv.3304.
